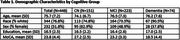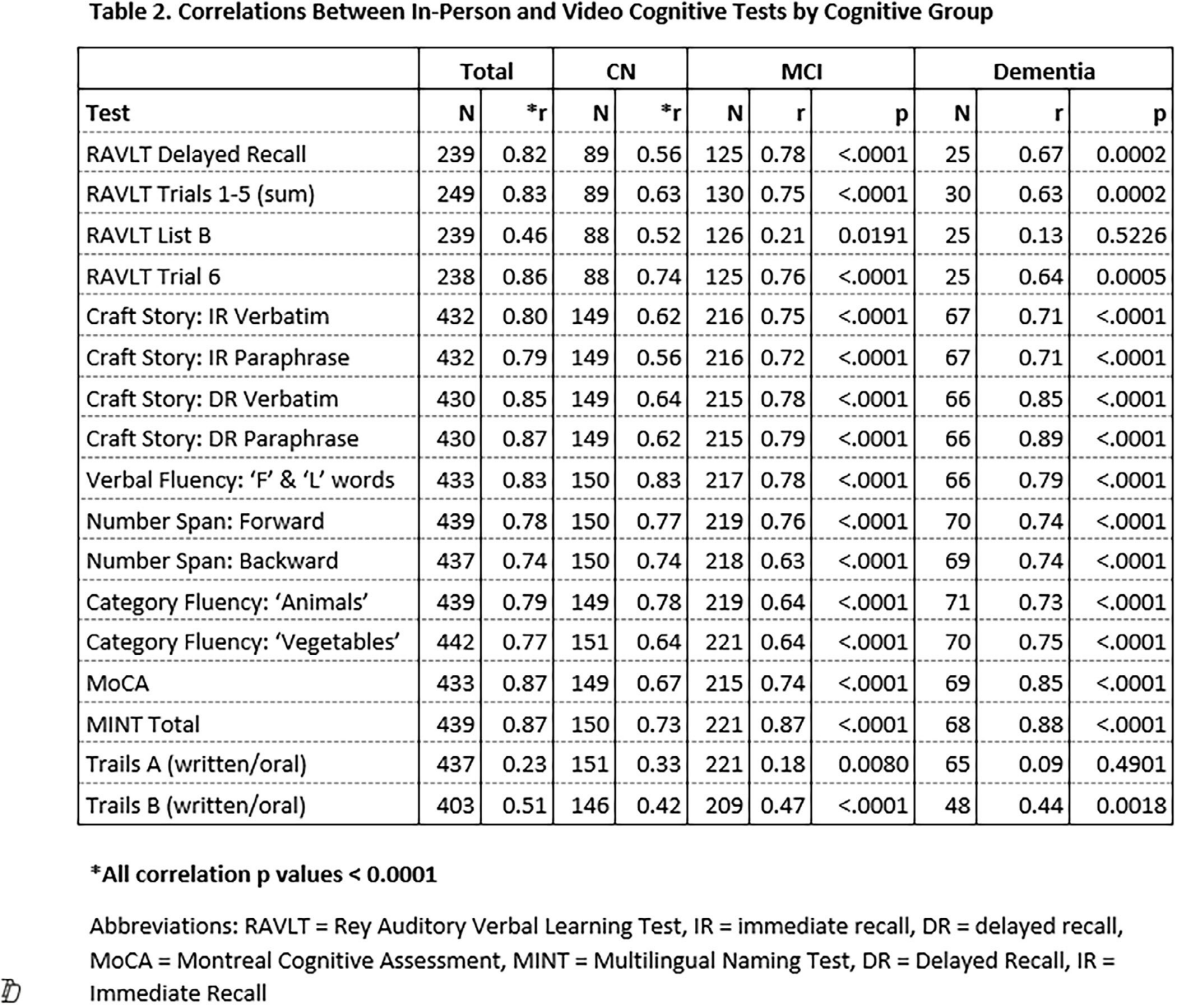# Validation of Video Administration of a Modified UDSv3 Cognitive Battery in Older Adults with and without Cognitive Impairment: The ‘VCog’ Study

**DOI:** 10.1002/alz70858_107261

**Published:** 2025-12-25

**Authors:** Bonnie C. Sachs, Lauren Latham, Suzanne Craft, Lindsay R Clark, Kevin Duff, Sarah Tomaszewski Farias, Felicia C. Goldstein, Benjamin M. Hampstead, Suman Jayadev, Gregory A Jicha, Walter W. Kukull, Xiaoyan Iris Leng, Dawn Mechanic‐Hamilton, Judith A. Neugroschl, Kathryn V Papp, Andrew J. Saykin, Margaret Sewell, Stephen R. Rapp

**Affiliations:** ^1^ Wake Forest University School of Medicine, Winston‐Salem, NC, USA; ^2^ Wake Forest School of Medicine, Winston Salem, NC, USA; ^3^ Wake Forest Alzheimer's Disease Research Center, Winston‐Salem, NC, USA; ^4^ Division of Geriatrics, Department of Medicine, University of Wisconsin School of Medicine and Public Health, Madison, WI, USA; ^5^ Oregon Health & Science University, Portland, OR, USA; ^6^ NIA‐Layton Aging & Alzheimer's Disease Research Center, Portland, OR, USA; ^7^ University of California, Davis School of Medicine, Sacramento, CA, USA; ^8^ Emory University School of Medicine, Atlanta, GA, USA; ^9^ University of Michigan, Ann Arbor, MI, USA; ^10^ University of Washington, Seattle, WA, USA; ^11^ University of Kentucky Sanders‐Brown Center on Aging, Lexington, KY, USA; ^12^ National Alzheimer's Coordinating Center, University of Washington, Seattle, WA, USA; ^13^ Department of Medicine, University of Washington School of Medicine, Seattle, WA, USA; ^14^ University of Pennsylvania, Philadelphia, PA, USA; ^15^ Icahn School of Medicine at Mount Sinai, New York, NY, USA; ^16^ Harvard Medical School, Boston, MA, USA; ^17^ Department of Radiology and Imaging Sciences, Indiana Alzheimer's Disease Research Center, Center for Neuroimaging, Indiana University School of Medicine, Indianapolis, IN, USA; ^18^ Wake Forest School of Medicine, Winston‐Salem, NC, USA

## Abstract

**Background:**

Video‐interfacing is increasingly being used in research and healthcare. However, the validity of video‐based cognitive testing has not been adequately established. The ‘VCog’ Study is directly comparing video‐based cognitive assessments to in‐person assessments administered to a diverse cohort of older adults with normal cognition (CN), mild cognitive impairment (MCI), and dementia.

**Method:**

448 participants (age (M=75.7 (7.2)), race (23% non‐white), sex (52% female), and education (M=16.5 (2.5)) from 12 Alzheimer's Disease Research Centers (ADRCs) around the country completed, in randomized order, a video‐adapted version of the Uniform Data Set Version 3 (UDSv3) from their home 4‐8 weeks before or after their annual in‐person cognitive assessment. A subset of participants also completed the Rey Auditory Verbal Learning Test (RAVLT). Participants were previously classified by their ADRC as CN, MCI, or mild dementia. Participants used their own device (if it had A/V capabilities and Wi‐Fi) or they were provided a loaned device. All participants were oriented to video procedures before testing and were tested independently.

**Result:**

Pearson correlation coefficients were generally high and statistically significant (rs .73‐.86; *p*s < .0001) for the overall sample on the RAVLT – Delayed Recall, Craft Story Immediate & Delayed Recall, Phonemic and Category fluencies, Number Span‐Forwards & Backwards, MoCA, and the MINT. A moderate correlation (*r* = .51, *p* = .0001) was noted for Trail Making Test‐Part B (TMT, comparing Written & Oral version). Weak relationships existed for TMT‐A (Written & Oral). Correlation strength varied slightly when separated by cognitive group but remained highly statistically significant, apart from non‐significant correlations for TMT‐A in the dementia group. Apart from TMT, test means did not differ by test modality (*p*s > .05).

**Conclusion:**

VCog is the first large, randomized, study of cognitively, ethnically and geographically diverse individuals to examine the validity of video‐administered UDSv3 cognitive battery relative to in‐person administration. Results show strong concordance between UDSv3 tests for individuals with and without cognitive impairment. Oral TMT‐A performed poorly due to being structurally different from written TMT‐A. Having a UDSv3 battery validated for video administration can help AD researchers reduce participant burden and increase accessibility to underserved populations. Funded by NIH/NIA R01 AG075959